# Interactive effect of tillage and crop residue management on weed dynamics, root characteristics, crop productivity, profitability and nutrient uptake in chickpea (*Cicer arietinum* L.) under Vertisol of Central India

**DOI:** 10.1371/journal.pone.0279831

**Published:** 2022-12-30

**Authors:** Kaushlendra Pratap Singh, Vasudev Meena, J. Somasundaram, Suchi Singh, Mohan Lal Dotaniya, Hiranmoy Das, Ompal Singh, Ajay Srivastava

**Affiliations:** 1 College of Agriculture, RKDF University, Bhopal, Madhya Pradesh, India; 2 ICAR-Indian Institute of Soil Science, Bhopal, Madhya Pradesh, India; 3 ICAR-Directorate of Rapeseed-Mustard Research, Bharatpur, Rajasthan, India; ICAR-Indian Institute of Pulses Research, APRC, Bikaner, INDIA

## Abstract

Tillage and crop residue management play an imperative role in soil physico-chemical properties that eventually affects crop productivity. The objective of the study to find out a compatible combination of tillage and crop residue management for achieving sustainable food production by improving soil properties, providing favorable environment to crop plants. Secondly, managing crop residues effectively to reduce environmental pollution arising due to crop residue burning. With this aim, a field experiment was conducted on six years continued running experiment under conservation agricultural practices during *rabi* season of 2019–20 on chickpea. The experiment was comprised of five tillage operations with or without crop residue in main plot and three levels of nutrients in sub plots laid out in split plot design with three replications. Reduced Tillage with 60cm residue height (RT60) was recorded higher growth and yield attributes over conventional tillage practice that attributed to economic yield enhancement. The percent yield increment under NT and RT with 30 and 60cm height residue retention varied from 6.91% to 9.67% over conventional tillage. Maximum grain (2380 kg ha^-1^) and biological output (5762 kg ha^-1^) was recorded under RT60 (T_4_), which ascribed to higher net return (Rs 60551 ha^-1^) and benefit-cost ratio (2.97). The augmentation in net monetary benefit among tillage systems was lies between 24.32% to 37.78% over conventional tillage. The seed protein content ranged between 20.38 to 21.69% among the treatments. Moreover, total N uptake was maximum under RT60, while total P and K uptake was higher in No Tillage with 30cm residue height (T_1_). No-Tillage with 60cm residue height (NT60) recorded relatively higher soil moisture content (SMC) (22.71 and 15.40%). Treatment NT30 accrued relatively higher value of soil bulk density (1.42 Mg m^-3^) followed by NT60 and RT60 in comparison to conventional tillage (1.34 Mg m^-3^). In conclusion, NT and RT with 60cm residue height along with STCR (N_3_) nutrient dose was found effective for sustainable food production.

## Introduction

India contributes to a major share of the world’s chickpea area (70%) and production (67%) and continues to be the largest chickpea-producing nation. Chickpea is a major pulse crop of India, accounting for more than 40% of the total pulses area and production, mainly grown as a rainfed crop (68% area). Chickpea recorded a highest ever production of 11.23 Mt, which was 46% of the total pulses production (23.95 Mt), with productivity of 1063 kg/ha from 10.56 Mha of area. Chickpea is a pre-dominant crop among pulses in Madhya Pradesh occupying 34% (35.90 lakh ha) of the total chickpea area and 41% (45.95 lakh tons) of total chickpea production in the country DAC&FW [1].

Long-term sustainability concerns are rising in agriculture as a result of over- and under application of fertilizers, intensive tillage, inadequate and improper resource management, causing soil health to deteriorate and crop production to decline [2, 3]. Therefore, environmental crisis, land degradation, and global food insecurity are the pressing concerns before the researchers and policymakers throughout the globe [4, 5]. Soil degradation, which is a major threat to sustainable food production system caused by inexpedient adverse farm practices like conventional tillage, crop residue removal or burning. The degraded soils having low soil fertility and low soil organic matter attributed to poor crop yield [6, 7]. Decline of soil physical properties like water holding capacity, soil structure, aggregate stability, increased soil erosion potential [8] eventually decreases crop productivity besides increasing environmental impairment [9]. Further, maintaining of soil health and crop productivity under this changing climate scenario is a challenging concern throughout the globe [10, 11]. Soil tillage plays a vital role in controlling soil properties such as temperature, moisture and soil bulk density which affect crop growth and productivity. Due to organic matter depletion in soils because of using heavy tillage operations, the agricultural system will not be sustainable based on conventional tillage. Additionally, conventional systems require lot of energy apart from destroying soil physical properties and its erodation. Further, shortage of irrigation water and an increasing price of fuel and fertilizers amplifying overall production cost and hence reducing profitability of production system. Studies reported that conventional agricultural practices like intensive tillage, imbalanced nutrition, residues burning etc. have accelerated food production cost by 4–5 times besides increasing energy use and greenhouse gas emissions substantially [12, 13]. Moreover, lack of moisture in the soil surface layers may cause plant to derive its moisture from deeper layers of the soil profile where essential nutrients are low and thus plant suffers from nutrient stress. Sum of these factors reduces plant size and existing photosynthetic reserves to fill the pods, and ultimately, it reduces the crop yield. Further, burning of crop residues in the field enhancing environmental pollution, greenhouse gas emission’s leading to global warming which is harmful for sustainable food production and environmental safety. Under such horrid situations, an effective management of tillage and crop residues may play crucial role.

Resource conservation technologies (RCTs) like zero tillage or minimum tillage with residue retention have emerged as a means of achieving the sustainability of intensive cropping systems [14]. In addition to reduction in the cost of cultivation (labour, fuel, farm equipment’s, land preparation), time saving and getting stable yields [15], RCTs also improve soil fertility through increased carbon accumulation and biological activity [16], and reduces energy inputs [17]. Water permeability in soil increases in low tillage systems due to increased organic matter and earthworm activity compared to conventional tillage system. Use of low tillage systems and soil freeze reduces the setbacks of conventional tillage like cost of energy consumption, soil erosion and degradation [18]. Soil surface under RCTs usually remains colder and wetter, and bulk density is higher than conventional tillage and this has an effect on growth of chickpea roots and absorption of nutrients.

On another side, recycling of crop residues left in the field after crops harvest, have advantage of converting the surplus farm waste into useful product for meeting nutrient requirement of crops. Studies reported that residue retention on soil surface maintain physical and chemical soil conditions [19] and improve overall ecological balance of the production system. Under current scenario, use of organic materials might be vital for sustainable production due to increasing cost of chemical fertilizers and to maintain soil health. Addition or retention of crop residue in the soil may play key role as a supplying source of nutrients for crop production and renovate soil physico-chemical properties and biological functions, if managed properly [20, 21], which increases the amount of food available to microorganisms, allowing for faster breakdown. The presence of sugar in organic wastes promotes decomposition and increases release of low molecular organic acids into the soil [10, 22]. Additionally, residue additions improve total nitrogen, carbon and other nutrients content in soil, which results in higher NPK uptake. Incorporation of organic N in soil as organic matter or crop residues improves soil fertility that substantially reduces requirement of fertilizer N and increases crop yield [23]. In zero or reduced tillage (RT), organic residues mostly remain on the soil surface resulting in reduced N mineralization [24]. Switching from conventional tillage (CT) to RT system, initially for a few years, may require more N application rates for sustaining crop productivity. A long term study (10 years) on soybean-wheat system in Vertisol at Bhopal (India) revealed that yield under NT and RT were equivalent to CT with reduced production cost and saving of energy and labour. Resource conservation technologies (NT and RT) coupled with residue retention or incorporation were as effective as conventional tillage in terms of crop productivity [25]. Somasundaram et al. [26] reported improved soil health and crop yield after five years of crop cycle under rainfed Vertisols of central India under conservation agriculture.

Considering all above facts, an investigation was under taken to study the effect of tillage practices and crop residue management combining with different nutrient levels on crop growth and productivity of chickpea in Vertisol of Central India with the hypothesis that a compatible combination of tillage and crop residue management might be an impactful strategy to improve the soil health and sustainable food production. Yet no information is available in this region on the feasibility of tillage in combination with crop residue and fertilizer management and their effects on soil health, crop growth, yield attributes and yield in chickpea. The explicit objectives of study were: (i). to assess the effect of different tillage practices and crop residue management on crop growth and yield component of chickpea under Vertisol and (ii) to evaluate effect of tillage and crop residue management on weed dynamics, soil physical properties and profitability.

## Materials and methods

### Description of experimental site and soil characteristics

The field experiment was carried out on the six years old continued running or existing experiment under conservation agricultural practices at ICAR-Indian Institute of Soil Science, Bhopal (India), during *rabi* season of 2019–2020. Soil of the experimental site is an Isohyperthermic, Typic Haplustert with deep heavy clayey in texture (24.5% sand, 23.5% silt and 52.0% clay), bulk density of 1.34 Mg m^–3^, 52.1% porosity, 0.83 mm mean weight diameter (MWD), 75.1 mm water stable aggregates (WSA), slightly alkaline in reaction (pH = 7.8), 4.5 g kg^–1^ organic carbon, 0.17 dS m^–1^ electrical conductivity. The soil was low in available alkaline KMnO_4_–N (86.5 g kg^–1^) and Olsen’ P (5.74 g kg^–1^) but high in available NH_4_OAc-K (222.3 g kg^–1^).

### Climate and weather conditions

**Fig 1:**
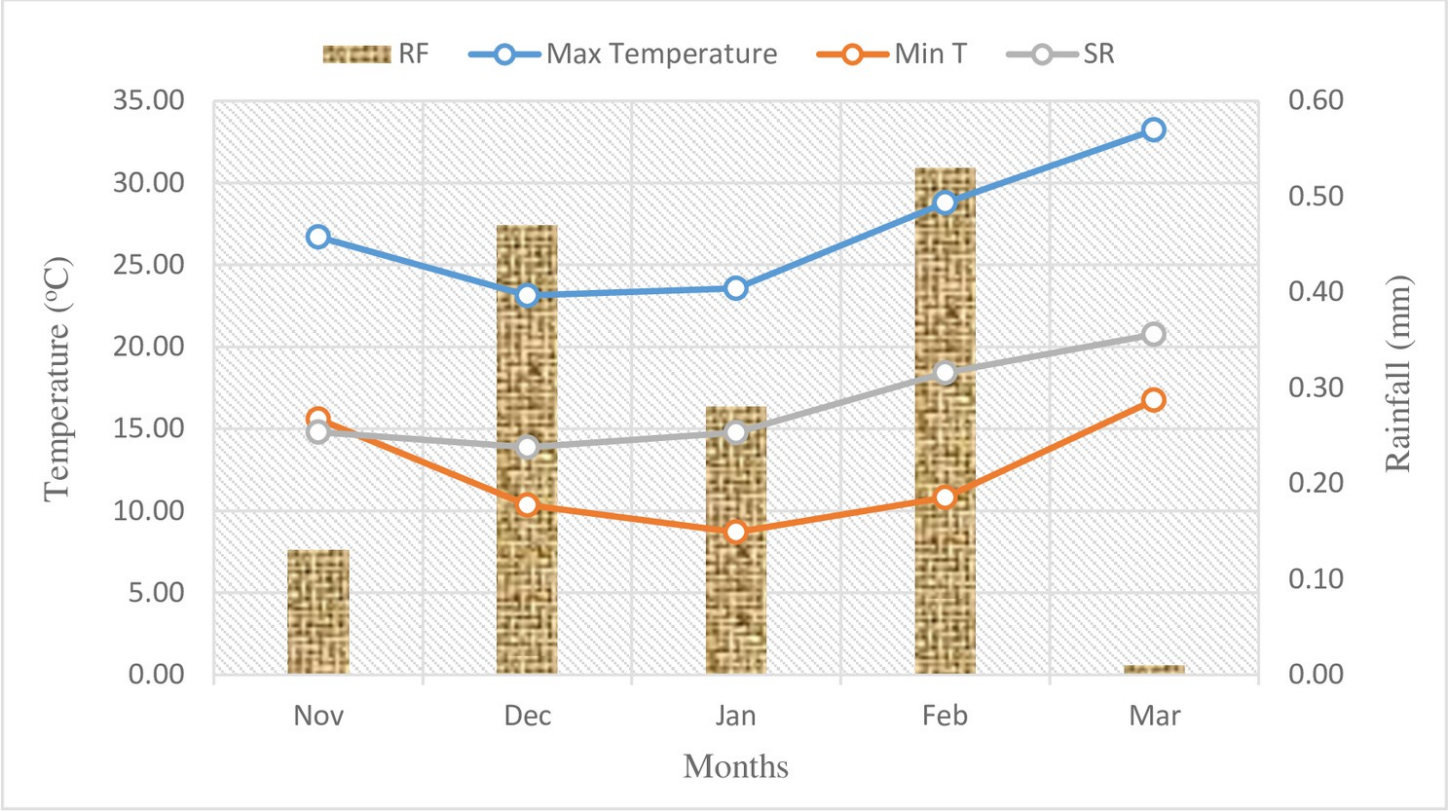
Monthly average rainfall, maximum and minimum temperature, solar radiation during crop growing season in Bhopal, India. (Max T: Maximum temperature (°C); Min T: Minimum temperature (°C); SR: Solar Radiation (Mj/m^2).

Geographically experimental site was situated at 23.10° N latitude and 77 20° E longitude with an altitude of 500m above the mean sea level. Climate of the region was typically humid sub-tropical region characterized by fairly cool and dry winter, hot and dry summer and warm and humid monsoon. The weather condition was congenial for satisfactory growth and development of chickpea and total rainfall during crop growth period was 1259 mm. The average of minimum and maximum temperature during the crop growth period of chickpea ranged between 18.2 to 33.6°C, respectively during 2019–20 (**Fig 1**). Similarly, the rainfall received during crop growing season was very less (1.42 mm only).

### Treatments and experimental design

The experiment was laid out in split plot design with three replications. The main plot treatments consisted of five tillage system *viz*. No Tillage (NT) with 30cm residue height (T_1_), No Tillage (NT) with 60cm residue height (T_2_), Reduced Tillage with 30cm residue height (T_3_), Reduced Tillage with 60cm residue height (T_4_), Conventional Tillage (T_5_) and sub-plot treatments consisted of three levels of nutrient doses as 75% RDF (N_1_), 100% RDF (N_2_) and STCR dose (N_3_). Under conventional tillage, the experimental field was prepared after pre-sowing irrigation. At proper moisture condition first ploughing was done with soil turning plough followed by two cross ploughing with cultivator. Whereas, no primary or secondary tillage operations were followed under no tillage treatments plots while less disturbances to soil is practiced in reduced tillage (one-time cultivation).

### Crop management and economic analysis

The chickpea variety JG-14 was used as test crop. Crop was sown in the second fortnight of November by following uniform sowing @ 80 kg/ha of seed using plot size of 6.0 m x 6.0 m. At sowing, a compound fertilizer (NPK) was applied at the rate of RDF (Recommended dose of fertilizer) 25:50:40 (N: P_2_O_5_: K_2_O kg/ha) and 30:80:25 (N: P_2_O_5_: K_2_O kg/ha) used under STCR. All the standard agronomic management practices like irrigation, weed management, pest and disease management etc. (recommended) were followed during crop growth period. The observations were taken on various growth parameters like plant population (no./m^2^), plant height, branches per plant, plant dry weight, root parameters (root nodules per plant and their dry weight, root length, root dry weight) at different growth stages and yield parameters like pod per plant, seed per pod, seed index, grain and biomass yield at crop harvest. Randomly five plants were selected from 2^nd^ rows of each plot for recording observations. One square meter area was marked at two places with the help of 1.0 x 1.0 m^2^ quadrate in each plot and number of plants coming within the marked area was counted. Mean values were expressed as the number of plant per square meter. Plant height from the ground level to top portion of the plant (in cm) with the help of meter scale was measured. Five plants were randomly selected and tagged properly. The leaves and stem from randomly selected plants of each plot and their roots were separated and shoot was weighed. For measuring dry weight per plant, samples were chopped into pieces, kept for sun drying and lastly in oven at 85°C till constant weight appear. Dry weight of the samples was averaged to get per plant weight. Root length from randomly selected five plants from each plot were taken out along with surrounding ball of soil with adequate care and washed gently in running water so as to avoid any type of damage to the roots. The root length was measured with the help of meter scale. Number of pods per plant, seed per pod and 1000 seed weight were measured and averaged value of each parameters was taken. The observations on weed composition, weed density and biomass were recorded using quadrats (0.5 x 0.5 m^2^) randomly placed at four places in each plot and then averaged it. The recovery of seed from total dry matter was considered as harvest index (HI) which was expressed in percentage and calculated by the following formula:

$$Harvest\;Index(\%)=\frac{Seed\;yield}{Biological\;yield}\times 100$$

### Chemical studies

Plant samples collected at harvest, were thoroughly washed with distilled water and dried at room temperature for 24 hours; oven dried at 65˚C till constant weight. The samples were ground to a homogenous powder using grinding machine and later on digested with H_2_SO_4_ and diacid (HNO_3_:HClO_4_ in 9:4 ratio) for estimating N, P and K, respectively. The uptake of nutrients was calculated from the concentration and respective seed/stover yield and total uptake was calculated by adding both stover and seed uptake. The nutrient uptake (N, P, K) by grain and stover was computed by the following relationship:

$$\begin{array}{l} Nutrient\;uptake\;(kg/ha)\\ \;=\frac{\%Nutrient\;in\;seed\;\times \;Seed\;yield(kg/ha)+\%Nutrient\;in\;straw\;\times \;Straw\;yield(kg/ha)}{100} \end{array}$$

#### Nitrogen uptake

Nitrogen content (%) of grain and stover was determined separately by Linder and Harley method [27], per cent nutrient both in grain and stover were multiplied with their respective yields and then were added to get total nitrogen uptake (kg/ha).

#### Phosphorus uptake

Phosphorus content in grain and stover were estimated by Vanadomolybdo-phosphoric yellow colour method as given by Olsen et al. [28], and there after total P uptake (kg/ha) was calculated.

#### Potassium uptake

Potassium content both in grain and stover was determined by Flame photometer method given by Toth and Prince [29], and thereafter total K uptake (kg/ha) was calculated.

*Protein content*. Per cent protein content in seed was calculated by multiplying the N content in seed with a factor of 6.25. Observations were also recorded on soil moisture content and soil bulk density under different tillage treatments.

Economic viability in terms of gross return, cost of cultivation, net return and benefit-cost ratio was worked out on the basis of present average market prices. Net revenue was calculated based on the economic value from grain-total inputs during crop production. The prizes of grain and all inputs were calculated according to the prevailing local condition. The total inputs included expenditures incurred on fuel for machinery, electricity for irrigation, labour charges, seeds, fertilizers and harvesting. The economic profit was calculated by equation [30].


$$Benefit\;-\;cost\;ratio(kg/ha)=\frac{Net\;return}{Total\;input}$$

### Statistical analysis

Data were subjected to the statistical analysis by using SAS 9.3. Analysis of variance was performed using PROCGLM after square root transformation (√x + 0.5) of the original data as appropriate for weed density and dry weight to hold the normality assumption, where, x is the observed value and 0.5 is a constant. The treatment means were separated at p = 0.05 using Tukey test.

## Results

### Weed flora

The experimental field was utterly invaded with dicots weeds. The weed flora under dicots includes species like *Parthenium hysterophorus* (38.81%), *Cirsium arvense* (31.12%), *Chenopodium album* (14.33%), *Melilotus indica* (8.39%) and *Anagallis arvensis* (7.35%) whereas, no monocots or grassy weeds were found in the field.

### Weed density and dry weight

Data on weed density and dry biomass was recorded under different treatment at 30, 60, 90 DAS and harvest stage during crop growth period. Number of weeds was significantly influenced by tillage at 30 and 90 DAS of crop only (Table 1). Data illustrated that maximum weed density (8.78, 9.11, 8.89 and 9.22) and dry weight (0.95, 1.49, 1.62 and 1.99 g) was recorded under NT30 (T_1_) at all growth stages. Whereas, minimum weed density (4.22, 3.67, 4.22 and 3.67) and dry weed biomass was recorded under T_5_ (conventional tillage) among all the treatments at different -time intervals (30, 60, 90 DAS and harvest stage) which was at par with treatment T_4_ and T_3_. Further, nutrient management treatments did not register any significant effect on weed density and dry biomass. However, maximum number of weed plants (6.87, 6.87, 6.93 and 6.93) and dry weight (0.90, 1.40, 1.51 and 1.85 g) was recorded under STCR (N_3_) followed by 100% RDF (N_2_), whereas N_1_ (75% RDF) registered lesser number of weed plants as well as weed dry biomass.

**Table 1:** Effect of tillage practices, crop residue management and nutrient levels on density and dry weight of weeds in chickpea.

Treatments	Weed density (no. m^-2^)				Weed dry weight (g)			
	30 DAS	60 DAS	90 DAS	At harvest	30 DAS	60 DAS	90 DAS	At harvest
Tillage								
NT with 30cm residue height (T_1_)	3.05a (8.78)	3.10a (9.11)	3.06a (8.89)	3.12a (9.22)	0.95 (0.41)	1.49a (1.72)	1.62a (2.14)	1.99a (3.45)
NT with 60cm residue height (T_2_)	2.80b (7.33)	2.80a (7.33)	2.82a (7.44)	2.82a (7.44)	0.89 (0.29)	1.33a (1.28)	1.55a (1.90)	1.77b (2.65)
RT with 30cm residue height (T_3_)	2.74b (7.00)	2.74a (7.00)	2.76a (7.11)	2.76a (7.11)	0.88 (0.28)	1.30a (1.20)	1.39a (1.44)	1.75b (2.58)
RT with 60cm residue height (T_4_)	2.30c (4.78)	2.17b (4.22)	2.32b (4.89)	2.20b (4.33)	0.87 (0.26)	1.27a (1.11)	1.37a (1.37)	1.76b (2.60)
CT (Conventional Tillage) (T_5_)	2.17c (4.22)	2.04b (3.67)	2.17b (4.22)	2.04b (3.67)	0.87 (0.25)	1.23b (1.01)	1.2b (1.15)	1.72b (2.46)
Nutrient levels								
75% RDF (N_1_)	2.51a (5.80)	2.48a (5.67)	2.52a (5.87)	2.50b (5.73)	0.88a (0.28)	1.25b (1.07)	1.40a (1.46)	1.74a (2.53)
100% RDF (N_2_)	2.66a (6.60)	2.60a (6.27)	2.69a (6.73)	2.63b (6.40)	0.89a (0.30)	1.33a (1.27)	1.42a (1.53)	1.81a (2.77)
STCR dose (N_3_)	2.71a (6.87)	2.71a (6.87)	2.73a (6.93)	2.73a (6.93)	0.90a (0.31)	1.40a (1.45)	1.52a (1.81)	1.85a (2.93)

*Data were subjected to square root transformation (√ x + 0.5), values in parentheses represent original values.

Different letters after values indicate significant treatment effect (Tukey’s test was used to check significant differences)

### Effect on growth attributes

The data on plant population and plant dry weight were significantly influenced by tillage system (Table 2). Among the tillage system, maximum plant population (36.0 no./m^2^) and plant dry weight (31.17 g) was recorded under RT60 (T_4_), while minimum (26.67 no./m^2^ & 23.51 g) were recorded under CT (T_5_) at harvest. The percent increase in plant dry weight were higher by 4.21, 9.82, 15.18 and 32.58% under T_1_, T_2_, T_3_ and T_4_ to the conventional tillage (CT) practice. Whereas, plant height and number of branches per plant were non-significant. With respect to nutrient management, all the treatments were found non-significant except number of branches per plant at harvest. However, the maximum values of growth attributes (plant population, plant height, branches/plant and plant dry weight) were found under N_3_ (STCR dose). The treatment N_3_ (STCR) was attained higher values of branches/plant (12.89) over N_1_ (75% RDF) and N_2_ (100% RDF).

**Table 2:** Effect of tillage practices, crop residue management and nutrient levels on plant growth attributes of chickpea.

Treatments	Plant population (no. m^-2^)	Plant height (cm)	Branches plant^-1^ (No.)	Plant dry weight (g)	Root nodules plant^-1^		Root parameters	
					Number	Dry weight (g)	Root length (cm)	Root dry weight (g)
Tillage								
NT with 30cm residue height (T_1_)	28.67bc	72.37a	12.11ab	24.50b	13.37b	0.05b	21.98ab	2.83b
NT with 60cm residue height (T_2_)	30.89abc	72.74a	12.70a	25.82b	14.11b	0.05b	23.96a	3.06ab
RT with 30cm residue height (T_3_)	34.33ab	72.74a	12.55ab	27.08b	15.56ab	0.07ab	24.21a	3.16ab
RT with 60cm residue height (T_4_)	36.00a	74.78a	12.78a	31.17a	18.63a	0.09a	24.42a	3.44a
CT (Conventional Tillage) (T_5_)	26.67c	72.18a	11.85b	23.51b	12.56b	0.06b	21.19b	2.75b
Nutrient levels								
75% RDF (N_1_)	29.40b	71.62a	12.04b	25.38a	13.29b	0.05b	22.08a	2.92a
100% RDF (N_2_)	32.07ab	73.20a	12.27b	26.13a	14.82ab	0.06ab	23.00ab	3.03a
STCR dose (N_3_)	32.47a	74.06a	12.89a	27.74a	16.42a	0.07a	24.38b	3.19a

RDF- Recommended dose of fertilizers, Different letters after values indicate significant treatment effect (Tukey’s test was used to check significant differences)

### Root characteristics

#### Root nodules per plant and their dry weight

The number of root nodules was found significant under various tillage systems (Table 2) and recorded maximum (18.63) under RT60. While, minimum number of root nodules (12.56) were observed under CT. The percent increase in the root nodule number by 4.21, 9.82, 15.18 and 32.58% achieved due to adoption of various tillage systems (T_1_ to T_4_) along with 30 or 60 cm height residue retention over the conventional practices. Nutrient management treatments were also ascribed significant effect on number of root nodules. Higher value (16.42) was found under STCR (N_3_) than other nutrient doses. Whereas, the dry weight of root nodules was non-significant for nutrient levels.

#### Root length and root dry weight

Effect of different tillage systems was non-significant on root length at harvest and recorded higher value (24.42 cm) under RT60 over the other tillage options (Table 2). At initial phases, the root length was minimum under NT30 (T_1_) but at later stages (at harvest) the least value was obtained under CT (21.19 cm). While in case of nutrient doses, the results were reversed to significant with respect to root length. At harvest, N_3_ -STCR registered maximum values of root length (24.38 cm). Data on root dry weight shows reverse trend of results compare to root length for both tillage and nutrient doses. Root dry weight was significant under different tillage system (2.90 to 25.09% higher) over conventional tillage whereas, it did not differ among the nutrient level.

### Effect on yield attributes, yield and profitability

#### Pod per plant, seed per pod and seed index

Data were recorded on yield attributing characters (pod plant^-1^, seed pod^-1^ and seed index) under different tillage systems and nutrient levels in chickpea. Results illustrated that pod plant^-1^, seed pod^-1^ and seed index were non-significantly influenced due to tillage system (Table 3). Maximum number of pod plant^-1^ (34.96), seed pod^-1^ (2.04) and seed index (14.31) were recorded under RT60 then rest of the treatments. While minimum values for pod plant^-1^ (33.33) was found under CT whereas, seed pod^-1^ (1.85) and seed index (13.82) obtained under NT30. Similar findings were also reported by Lanca rodrigues et al. [31] and indicated higher values of seed per pod under reduced tillage than under no tillage. Nutrient management treatments significantly influenced seed pod^-1^. Nutrient dose STCR (N_3_) recorded significantly higher seed pod^-1^ (2.00), pod plant^-1^ (34.53) and seed index (13.99) which was significantly higher than N_1_ (1.85, 33.51 and 13.92) and N_2_ (1.96, 34.20 and 13.97).

**Table 3:** Effect of tillage practices, crop residue management and nutrient levels on yield attributes, yield and economics of chickpea.

Treatments	Pods plant^-1^	Seed pod^-1^	Seed index	Yield (kg ha^-1^)			HI (%)	Profitability (Rs ha^-1^)			BC ratio
				Grain	Stover	Biological		Gross return	Cost of cultivation	Net return	
Tillage											
NT with 30cm residue height (T_1_)	33.63ab	1.85a	13.82b	2165b	3056b	5221c	41.47a	73726b	19089b	54636b	2.86b
NT with 60cm residue height (T_2_)	34.07ab	1.92a	13.89b	2320a	3125a	5445b	42.61a	78836a	19089b	59747a	3.13a
RT with 30cm residue height (T_3_)	34.41ab	1.92a	13.93b	2370a	3217a	5587ab	42.42a	80398a	20339b	60059a	2.95b
RT with 60cm residue height (T_4_)	34.96a	2.04a	14.31a	2380a	3383a	5763a	41.30a	80890a	20339b	60551a	2.97ab
CT (Conventional Tillage) (T_5_)	33.33b	1.92a	13.83b	2170b	3540a	5710a	38.00b	73936b	29989a	43947c	1.46c
Nutrient levels											
75% RDF (N_1_)	33.51b	1.85b	13.92a	2121b	3127b	5248c	40.42a	72254b	21145b	51109b	2.53b
100% RDF (N_2_)	34.20ab	1.96ab	13.97a	2322a	3280a	5602b	41.45a	78894a	21627b	57267a	2.77a
STCR dose (N_3_)	34.53a	2.00b	13.99a	2399a	3386a	5785a	41.47a	81523a	22536a	58987a	2.73a

RDF- Recommended dose of fertilizers, Different letters after values indicate significant treatment effect (Tukey’s test was used to check significant differences)

### Yield and harvest index

Data on grain, stover and biological yield (kg ha^-1^) were significantly differ among the tillage system. The percent yield increment under NT and RT with 30 and 60 cm height residue retention varied from 6.91% to 9.67% over conventional tillage system (Table 3). Maximum grain yield ha^-1^ (2380 kg) and biological yield ha^-1^ (5762 kg) was recorded under RT60 while, maximum stover yield (3540 kg ha^-1^) was obtained under CT. Lowest value of grain yield (2165 kg ha^-1^) under NT30 which was at par with the CT. Similarly, the lesser values of stover yield (3056 kg ha^-1^) and biological yield (5221 kg ha^-1^) respectively were recorded under NT30.

Nutrient management treatments were also affected grain, stover and biological yield significantly. The percent variation in the yield lies between 9.47% to 13.10% over the N_1_ (75% RDF). Highest grain yield (2399 kg ha^-1^), straw and biological yield (3386 and 5785 kg ha^-1^) were achieved under STCR dose (N_3_) while lowest grain (2121 kg ha^-1^), stover (3127 kg ha^-1^) and biological yield (5248 kg ha^-1^) found under N_1_ (75% RDF). Maximum value of harvest index (42.61%) was recorded under NT60 followed by RT at 30 and 60 cm residue height (T_3_ and T_4_). Under nutrient management, higher values of harvest index (41.47%) recorded under STCR (N_3_) as compared to other treatments of N_1_ and N_2_ (40.42 and 41.45%).

### Profitability

Results indicated that higher cost of cultivation was recorded under CT (T_5_) (Rs 29989 ha^-1^) followed by RT30 and RT60 (Rs 20339 ha^-1^ each) (Table 3). Higher net return was fetched under RT60 (Rs 60551 ha^-1^) and RT30 (Rs 60059 ha^-1^) followed by NT30 and NT60 (Table 2) whereas, lowest net return was obtained under CT (Rs 43947 ha^-1^). The percent increment in net monetary benefit among the different tillage systems varies from 24.32% to 37.78% with respect to conventional tillage practices. Residue application generated significantly higher income due to better soil fertility which augmented the yields and returns, although the part of returns was reduced by the cost of crop residues. Similarly, under nutrient management practices, STCR (N_3_) recorded higher net monetary return (Rs 58987 ha^-1^) which was higher by 3.0% and 15.41% over N_2_ and N_1_. The economic analysis of treatments in term of B-C ratio revealed that all the treatment significantly affected benefit-cost ratio. Alike, net return, higher B-C ratio was obtained under NT60 (3.13) which is at par with RT30 (2.95) next in order. Similarly, lower B-C ratio was registered under CT (1.46). Furthermore, N100% RDF (N_2_) recorded highest B-C ratio (2.77) among the nutrient management practices than other treatments.

### NPK uptake by seed

Data pertaining to N, P and K uptake by seed of chickpea showed significant influence by the different tillage system and nutrient levels (Table 4). Maximum seed N uptake (82.76 kg ha^-1^) was found under RT60 which was at par with the CT then rest of the treatments. While, P (7.03 kg ha^-1^) and K uptake (29.61 kg ha^-1^) was higher under NT30 as compare to other treatments but did not differ significantly among the tillage treatments. Minimum P-uptake (6.31 kg ha^-1^) was recorded under RT60 and K-uptake (26.20 kg ha^-1^) under NT60, respectively. Under nutrient management maximum NPK uptake (85.40, 6.97 & 29.20 kg ha^-1^) was obtained under STCR (N_3_) followed by N_1_ and N_2_.

**Table 4:** Effect of tillage practices, crop residue management and nutrient levels on nutrient uptake of chickpea.

Treatments	Nutrient uptake seed (kg ha^-1^)			Nutrient uptake stover (kg ha^-1^)			Total nutrient uptake (kg ha^-1^)		
	N	P	K	N	P	K	N	P	K
Tillage									
NT with 30cm residue height (T_1_)	78.86bc	7.03a	29.61a	15.38a	8.62a	57.79ab	94.24a	15.65a	87.40a
NT with 60cm residue height (T_2_)	74.31d	6.36a	26.20c	13.53b	7.57b	52.74c	87.84b	13.93b	78.94c
RT with 30cm residue height (T_3_)	76.20cd	6.53a	26.47bc	14.05ab	8.24a	53.82bc	90.25b	14.77ab	80.29bc
RT with 60cm residue height (T_4_)	82.76a	6.31a	29.03ab	14.43ab	8.17ab	55.17abc	97.19a	14.48ab	84.20ab
CT (Conventional Tillage) (T_5_)	79.75ab	6.86a	27.29ab	15.10a	8.20ab	58.10a	94.85a	15.06ab	85.39a
Nutrient levels									
75% RDF (N_1_)	68.29b	6.16b	25.47b	13.34b	7.52b	51.94b	81.63b	13.68b	77.41b
100% RDF (N_2_)	81.43a	6.73ab	28.49a	15.14a	8.42a	57.33a	96.57a	15.15a	85.82a
STCR dose (N_3_)	85.40a	6.97a	29.20a	15.00a	8.54a	57.29a	100.4a	15.51a	86.49a

Different letters after values indicate significant treatment effect (Tukey’s test was used to check significant differences

### NPK uptake by stover

Effect of tillage system on N and K uptake was significant while it was non-significant for P uptake (Table 4). Maximum N and P uptake (15.38 & 8.62 kg ha^-1^) by straw was observed under NT30, while, K uptake was maximum (58.10 kg ha^-1^) under CT. Minimum values of NPK uptake accounted under NT60. Further, in case of nutrient management treatments maximum N (15.14 kg ha^-1^) and K-uptake (57.33 kg ha^-1^) was measured under 100% RDF (N_3_) whereas, maximum P-uptake (8.54 kg ha^-1^) was obtained under STCR (N_3_) followed by N_1_ (13.34, 7.52 & 51.94 kg ha^-1^).

### Total nutrient uptake

Data from the table illustrated significant effect of tillage and crop residue management as well as nutrient levels on total NPK uptake (Table 4). The similar trend of NPK as seen in seed and stover, was observed in case of total uptake both for conservation tillage practices and nutrient levels.

### Soil moisture content

**Fig 2:**
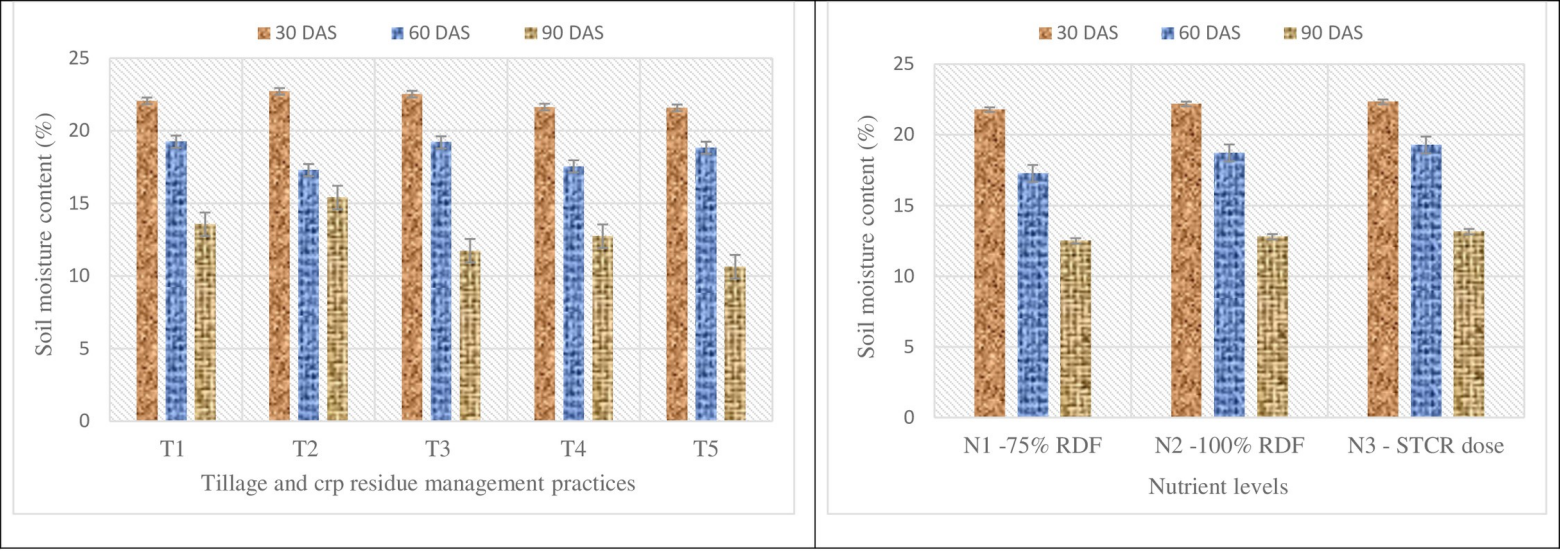
Effect of tillage practices, crop residue management and nutrient levels on soil moisture content. (T_1_—NT with 30cm residue height; T_2_—NT with 60cm residue height; T_3_—RT with 30cm residue height; T_4_—RT with 60cm residue height; T_5_–Conventional Tillage).

Data on soil moisture content (SMC) was significant under tillage system at 90 DAS of crop only than other two time intervals (Fig 2). Data depicted that no tillage system performed better with respect to soil moisture conservation than reduced tillage either at 30 or 60cm residue height and conventional tillage practice. Among tillage system, NT60 recorded relatively higher SMC value (22.71 and 15.40%) at 30 and 90 DAS. Whereas, NT30 recorded maximum SMC value (19.25%) at 60 DAS over the other treatments. Lowest SMC value (21.58 and 10.64%) was observed under CT at 30 and 90 DAS. Nutrient levels did not register any significant effect on SMC. However, highest value of SMC (22.23, 19.28 and 13.15%) was observed in N_3_ (STCR) at all growth stages of crop (30, 60 and 90 DAS).

### Soil bulk density

**Fig 3:**
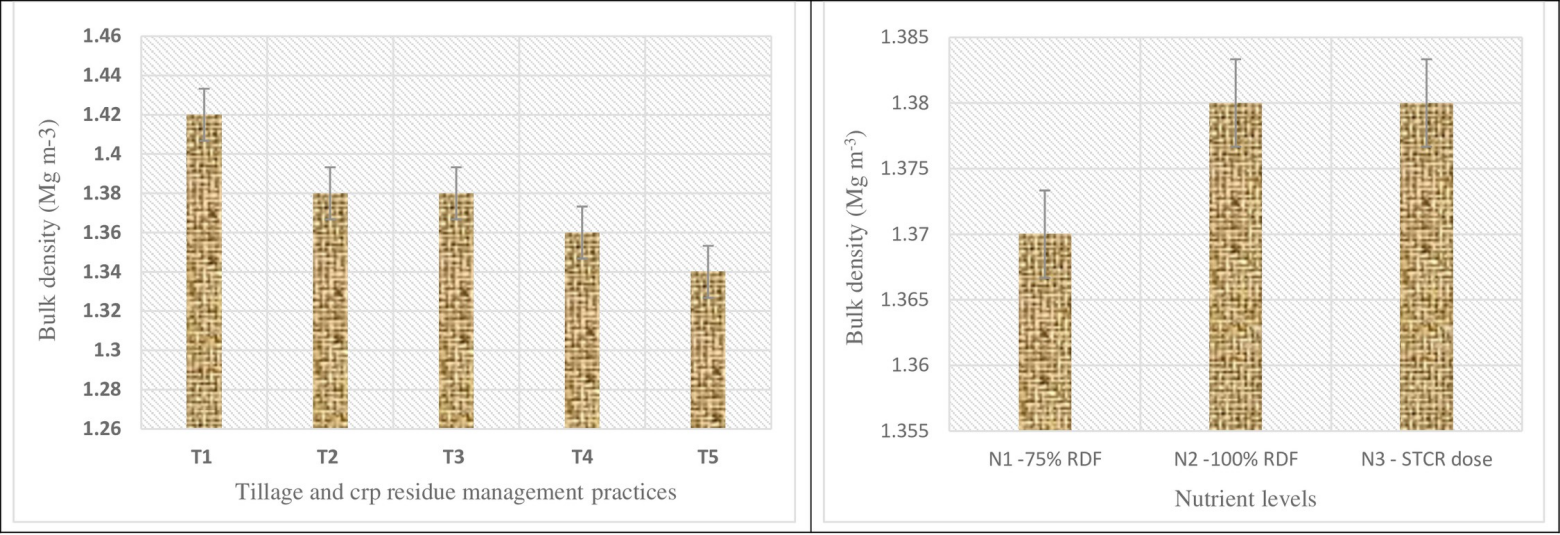
Effect of tillage practices, crop residue management and nutrient levels on soil bulk density. (T_1_—NT with 30cm residue height; T_2_—NT with 60cm residue height; T_3_—RT with 30cm residue height; T_4_—RT with 60cm residue height; T_5_–Conventional Tillage).

Data pertaining to soil bulk density (SBD) illustrated that tillage system has significantly influenced the SBD while, nutrient levels were found non-significant (Fig 3). Likewise, SMC, similar trend of response among the tillage system was observed for SBD. Amongst tillage treatments, highest SBD value was observed under NT30 (1.42Mg m^-3^) followed by NT60 and RT60, whereas lowest value of SBD was recorded under conventional tillage (1.34 Mg m^-3^). Nutrient management treatments did not affect SBD significantly and values falls between the range of 1.37–1.38 Mg m^-3^.

### Protein content in seed

**Fig 4:**
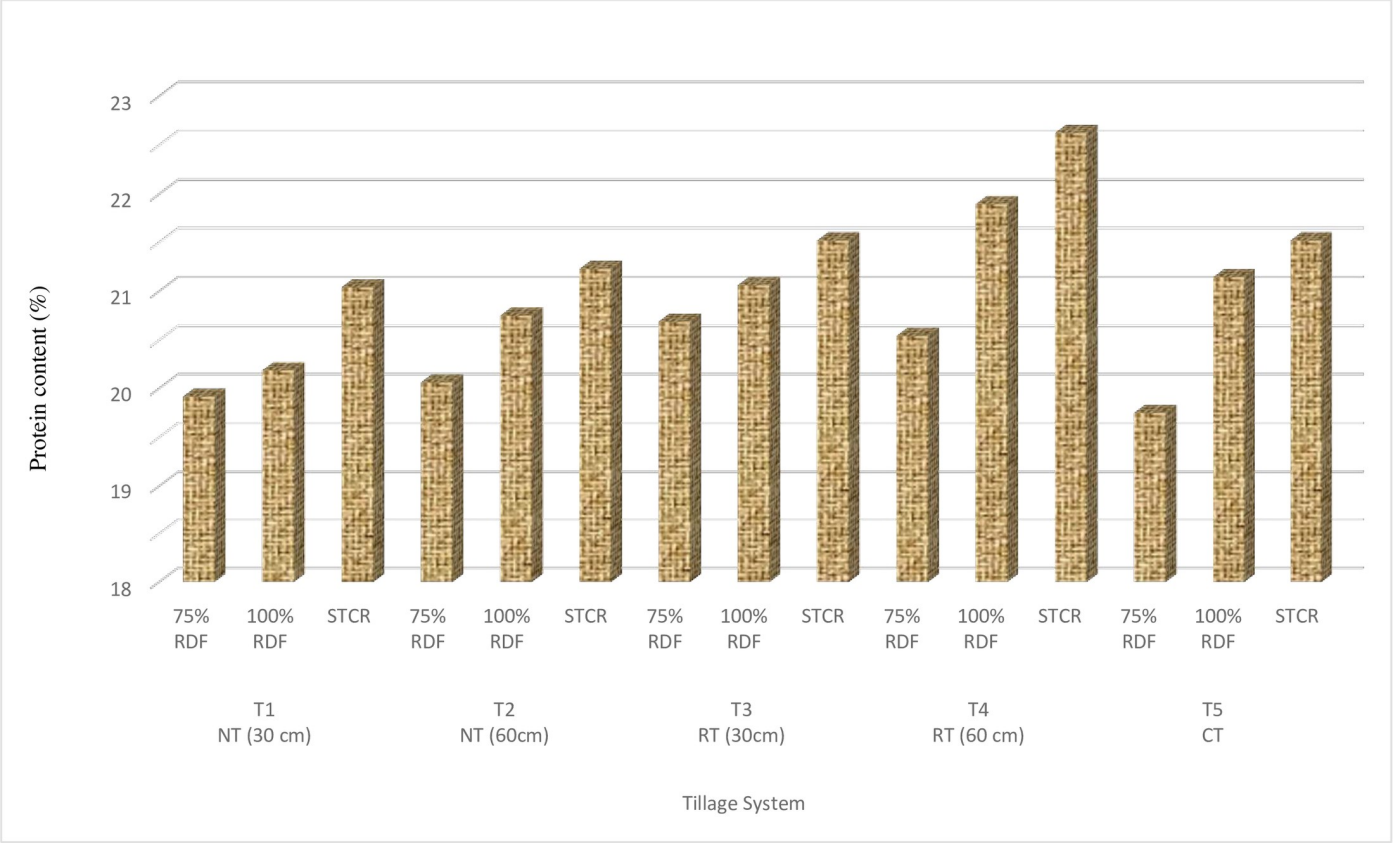
Effect of tillage practices, crop residue management and nutrient levels on seed protein content. (T_1_—NT with 30cm residue height; T_2_—NT with 60cm residue height; T_3_—RT with 30cm residue height; T_4_—RT with 60cm residue height; T_5_–Conventional Tillage).

The data indicates significant differences in seed protein content due to application of various tillage and nutrient management treatments. The seed protein content ranged in between 20.38 to 21.69% (Fig 4). However, highest protein content (21.69%) obtained in RT60 followed by other treatments while minimum value (20.38%) was observed under CT. Under sub-treatments highest protein content (21.59%) was recorded under STCR (N_3_) that was at par with 75% and 100% RDF.

## Discussion

Weed density and species richness on aboveground were found higher under continuous no-till (NT) than conventional tillage (CT). Higher weed population in ZT might be due to the presence of more weed seeds on soil surface, which may promote greater and quick emergence of weed species that require light to germinate or smaller seeds that cannot emerge after burial by tillage [32, 33]. Similarly, retention of crop residues on the soil surface reduced weed germination and density as compared to crop removal plots, but crop residue did not affect weed dry matter accumulation. Surface residue retention modifies soil properties such as temperature, moisture, and light intensity and quality that ultimately changed emergence behavior of some sensitive weed flora [34]. Continuous residue retention over three years’ period reduced total weed density and dry matter i.e. plots with residue accumulate 16.7% less dry matter than plots without residue [35]. Surface residue retention in zero-tillage suppresses weed emergence to a certain extent, residues also restrict manual or mechanical weed control [36]. The experimental results were also corroborated with the findings of Chaudhari et al. [37], reported highest dry biomass of total weeds under zero tillage followed by zero tillage + residue treatment.

The yield is fraction of total biomass (total dry matter accumulation) that available in the form of economic yield (grain) which is the ultimate result of bio-physiological processes. This is reflected by the source-sink relationship. The yield attributing characters (pod plant^-1^, seed pod^-1^, seed index) were increased non-significantly under different treatments at various growth stages. Data on yield attributes were relatively higher in the minimum tilled plot than untilled (NT) and conventionally tilled plots [38]. Number of pods were reduced by 5.88% as compared to reduce tillage which is due to the tillage effect on physical and chemical properties of soil. Seed index is a quality parameter to assess grain quality and this trait is generally affected by genetic makeup of varieties and application of inputs to the crop. Maximum value of seed index was recorded under RT60 (T_4_) and STCR (N_3_) then rest of the treatment and sub-treatments. This might be attributed to improved physico-chemical properties of the soil, increased carbon concentration creating favorable conditions for crop growth, consequently leading to efficient photosynthesis and translocation of photosynthates from source to sink resulting in increased crop biomass. Hence, higher total biomass yield under residue addition could have led to significant increase in crop productivity [39]. Schwab et al. [40] indicated that conventional tillage eliminated compaction of sub-surface soil due to deep tillage, which may have enhanced root growth and subsequent nutrient and water uptake thereby produced higher seed yield. Getting of higher yield is also result of effective weed control that enhanced yield attributes and thereby yield [37].

Crop residue retention treatments had higher yields than that of crop residue removals, suggesting that field mulching with crop residue promotes soil health and crop productivity. This is because of residues and their decomposition improves the soil structure through enhancing soil aggregate stability and soil properties while limiting soil water evaporation and soil crusting [41]. The improved soil properties increased its water infiltration and water availability to the crop [42, 43], which enhances the crop output. Additionally, mulching with crop residues impacts weed emergence and biomass, so that competition between weeds and crop could be mitigated and crop yield may increase [44]. Ojeniyi and Adekayode [45] reported similar result for maize and cowpea yield. Similarly, Di Ciocco et al. [46] observed significantly higher production per unit area under conservation tillage system compared with conventional tillage. Different tillage systems exerted similar soil conditions and thereby created equal influence upon crop yield, straw yield, harvest index and seed index. Winter and Unger [47] confirmed that no tillage produced paramount dry biomass over other tillage treatments but harvest index was not different across the treatments. Low dry matter accumulations in zero tillage, possibly due to compact soils layer at plough depth in the initial years, and which might have adverse effect on root and plant growth [48, 49]. In rice-maize system, maize yield was significantly reduced by 7.9% when no residue was applied to previous crop (rice), while yield was improved by 8.2% when residue applied @ 3 t/ha [50].

Getting maximum profitability lies not only in reducing use of input per unit area but also in lowering costs per unit crop production through higher yields. Using higher number of input and other field operation under conventional tillage practices, increases cost from the above treatments while less cost of cultivation under conservation tillage increases net monetary benefit. Adoption of no-tillage practice declined the cost of cultivation by 35% for direct-seeded upland rice over conventional tillage [50]. Similar findings (25–30% less cost of production) were also reported by Lal et al. [4]. Suryavanshi et al. [51] reported that zero-tillage with residue retention of preceding crops recorded maximum N, P and K in green gram. The zero tillage + residue treatment has accumulated maximum available phosphorus and potassium compared to rest of the treatments [52]. Moreover, residue burning and soil tillage under conventional agriculture result in the loss of valuable soil moisture. Korucu et al. [53] illustrated that the burning of wheat stubble resulted in a rapid moisture loss especially in upper 10cm of the soil profile, and the total amount of loss was 41%. Soil water content on sandy clay loam was higher under conservation tillage than conventional tillage [54]. SMC in surface soil is always higher in ZT with residue retention than CT due to less water evaporation, radiation insulation effect of residue and shedding effect [55]. Available soil water contents in the top 12 cm soil under residue retention and no-till were higher than those under residue burned and tilled treatments [56]. A higher evapotranspiration (ET) in NT plots than in CT and RT plots has also been reported that attributed to greater and deeper soil water storage [57] as extensive tillage usually exposes soil surface to water loss and evaporation. Zero tillage exhibited slightly higher BD than conventional tillage because of less porosity in surface soil as no ploughing was done. Whereas, in subsurface soil, ZT recorded considerably lower BD than CT due to using of less machinery (machine weight, tyre width, inflation pressure), number of passing, as well as optimum soil moisture content [58]. Addition of crop residue to the soil surface reduces its bulk density because residue accumulates in the surface soil and due to more macro-pores development and better soil aggregation. Shaver et al. [59] reported that each ton ha^-1^ crop residue addition over a 12-year period reduced bulk density by 0.01 Mg m^-3^. Similar results were also found by others researchers [51, 60].

## Conclusion

Land preparation alone contributes about 25–30% of production cost which can be reduced by adopting conservation tillage in comparison to conventional practices. CA based modified tillage and appropriate residues management can produce both immediate and long-term benefits like improved soil quality with sustainable and profitable productivity. Based on experimental results, it can be concluded that after six years of continued conservation tillage, reduced tillage with 30 and 60cm residue height was found more effective than conventional tillage followed by no tillage at both residue heights. The crop growth parameters, yield attributes and yield was significantly influenced by adopting RT60 (T_4_) and RT30 (T_3_), which ascribed to maximum net monetary benefit apart from conserving soil moisture, increased NPK uptake and reduced weed density and dry biomass. Further, retention of crop residue either in RT or NT with 30 or 60cm height in the field improved soil health. The application of RT or NT practices is eco-friendly, environmentally safe and economically viable as it saves time and money as compared to conventional tillage. The STCR based nutrient management (N_3_) was found superior in improving crop performance and NPK uptake. Overall, a combination of NT and RT with 60 cm residue height along with STCR (N_3_) nutrient dose was performed better for sustainable food production. The technology might be helpful in reducing cost of production by reducing labour requirements, fuel charges, irrigation and fertilizer needs with improved soil health, sustainable yield and clean environment.
